# Irresponsible parties, responsible voters? Legislative gridlock and collective accountability

**DOI:** 10.1371/journal.pone.0229789

**Published:** 2020-03-02

**Authors:** Asger Lau Andersen, David Dreyer Lassen, Lasse Holbøll Westh Nielsen

**Affiliations:** 1 Department of Economics and Center for Economic Behavior and Inequality, University of Copenhagen, Copenhagen, Denmark; 2 Department of Economics, University of Copenhagen, Copenhagen, Denmark; Sogang University (South Korea), REPUBLIC OF KOREA

## Abstract

Legislative gridlock is a failure of one of the key functions of government: to pass legislation. Can voters counter such political dysfunction? This paper examines whether and how voters hold politicians accountable for gridlock. We focus on the passage of the government budget, the central task of any legislature, and define a legislature to experience budgetary gridlock if it fails to pass the budget on time. We argue, based on evidence from twenty years of budget enactment data, that voters hold state legislators accountable for budget gridlock in US state governments, with gridlocked incumbents losing their seat more often than incumbents passing budgets on time. Based on established theories of party organization in American politics, we develop three competing theoretical hypotheses to guide our understanding of the observed patterns of retrospective voting. We find strong support for collective electoral accountability with voters punishing incumbent members of state legislature majority parties.

## Introduction

Legislative gridlock, the inability of legislative bodies to pass legislation, is a key concern of democratic politics. In December 2018, the US federal government shut down when lawmakers failed to reach agreement on a budget or, at least, a continuing resolution. The shutdown lasted 35 days, making it the longest in US history, and led to around 800,000 federal workers being furloughed or working without pay [1]. Brief federal government shutdowns also occurred in January and February of 2018, while the federal shutdown of 2013 lasted 16 days and caused significant economic disruptions.

LegisIative gridlock also affects state governments: in 2009, the state of California had to resort to Registered Warrants (popularly known as IOUs) to cover payments, passing a budget 24 days into the fiscal year; in 2010, the California budget was more than three months late.

Can voters hold lawmakers responsible for such lack of legislative productivity, or does gridlock constitute a system failure that electoral accountability cannot solve? Little is known about the electoral consequences of gridlock, both theoretically and empirically, partly due to disagreement over its measurement [2–4], partly due to a narrow focus on the case of the US Congress [4].

In this paper, we focus on electoral accountability for a specific set of gridlock instances: budget delays in US state governments. Although it does not encompass all types of legislative gridlock, this measure has several attractive features as an empirical operationalization of the broader phenomenon: first, the budget is the key piece of legislation for any legislature [5], and certainly so for US state legislatures [6]. Failure to pass a budget on time often has visible consequences for citizens: while state legislatures in some cases pass temporary budgets allowing limited appropriations, other cases result in a shutdown of all non-essential services and interrupted payments to agencies, employees and state contractors. Second, budget promptness, argued to be a key indicator of good government [7], rarely favors one party or political persuasion over the other.

Our measure of budget gridlock has two methodological advantages. First, any measure of gridlock should reflect both the supply and demand for legislation [2]: while legislative passage of a limited number of bills could reflect gridlock, it could alternatively reflect a lack of demand for legislative action. Our proposed measure does not suffer from this problem, as the budget must be passed at regular intervals. Second, budget lateness is a conceptually simple and replicable measure of legislative gridlock, and it is comparable across states as well as time.

We analyze voter responses to budget gridlock by comparing individual-level electoral outcomes for incumbent state legislators overseeing late budgets vs. those who manage to pass the state budget on time, controlling for a range of economic and political controls, as well as state and year fixed effects. The assumption necessary for strict causal identification is that, conditional on these controls, budget lateness is uncorrelated with other factors affecting state legislative outcomes. A relevant concern here is that budget lateness may correlate with (unobservable) gridlock over other issues. Indeed, as explained above, one may think of the budget delay variable as a proxy for legislative gridlock more broadly. Thus, a perhaps more reasonable assumption is that budget delays are conditionally uncorrelated with outcome-relevant factors *not directly related to legislative productivity*. Under this milder assumption, our estimates will not identify the causal effect of late budgets per se, but they *will* reflect the electoral consequences of legislative gridlock in a broader sense. Given our extensive battery of controls, we believe this assumption is quite plausible.

Because passage of the budget involves many different players, from the executive as well the legislative branches of government [5, 8], we argue that it is à priori unclear who voters will hold accountable for budget gridlock, if any at all. Based on the empirical approach described above, we find that voters do hold legislators accountable for legislative gridlock to some extent, but only those legislators who belong to the majority party in the state legislature. Analyzing more than 24,000 individual electoral races spread across 242 lower-house elections in 31 US states, we find that the reelection rate for majority party incumbents overseeing gridlocked budgets is 2–4 percentage points lower than for those who oversee timely budget passage. Although modest in size, this difference is statistically highly significant and robust to inclusion of a long list of controls. We find no such difference for minority party incumbents. We also demonstrate that a key institutional feature of the budget process–whether the government shuts down or can continue operations in the case of a late budget–is an important factor for understanding the electoral response to legislative gridlock.

Despite their modest size, our estimates suggest that about one fifth of state legislative elections are sufficiently close that whether the state budget is passed on time or not could be decisive for which party will gain control over the state legislature. This is remarkable, given that the literature on state legislative elections has struggled to identify any within-state factors beyond incumbency that actually affect electoral outcomes [9–11]. We believe this lack of significant results is partly due to a focus on economic outcomes for which the executive is typically held responsible [9, 12], or on individual legislator actions that receive little media coverage [11]. Thus, one interpretation of our results is that voters attribute responsibility to majority party members of the state legislature for *collective* outcomes that they can in fact affect–such as the timely passage of a budget–rather than for individual legislator actions or outcomes outside the legislature’s direct control.

Our paper explores individual accountability for collective performance in American politics. Early contributions to this literature [13, 14] argued that the declining role of parties in American politics meant that members of the US Congress faced little or no accountability for their performance in office: if parties are weak, an incumbent's partisan affiliation carries little information to voters, and incumbents can escape responsibility for poor collective performance by distancing themselves from their own party, and empirical evidence from the US House of representatives shows that recent increases in party unity and partisan polarization has strengthened accountability [15, 16].

These studies measure accountability as voter reactions to some measure of voters' subjective evaluation of congressional performance, but they do not examine how these evaluations depend on actual legislative output. Other research focuses on the link from legislative output and governance, in the form of late budgets and ethics violations, to voter evaluations [17]. In contrast to both approaches, our paper examines the direct link from observed legislative performance to actual electoral outcomes.

At the same time, our results can be seen as providing empirical support for some fundamental–but empirically untested–assumptions in Cox and McCubbins's influential procedural cartel theory [18, 19]. The basic premise of this theory is that the reelection prospects of individual majority-party incumbent legislators depend on voters' evaluations of the "brand" of their party, which in turn depend on the party's legislative performance. Existing empirical evidence [15] considers the first part of this premise, i.e. the link from party brand to reelection prospects, whereas our results also extend to the second part, that is, the link to actual legislative performance.

Finally, the paper speaks to the causes and consequences of legislative productivity. Mayhew [2] rejected the conventional wisdom that divided government hinders passage of important legislation, whereas a number of later studies have found evidence consistent with the conventional view (e.g. [3, 4, 20–22]). To our knowledge, the only study to analyze the electoral *consequences* of (lack of) legislative productivity is Binder [4] who examines the effect of legislative gridlock in Congress on the electoral fortunes of House members and finds no statistically significant relationship. It is not clear to what extent these results for Congress reflect limited statistical power, nor whether they generalize to legislatures at other levels of government.

## Gridlock and electoral accountability: Three hypotheses

In this section, we formulate three competing hypotheses about the electoral consequences of budget gridlock in state legislative elections. Common to all three hypotheses is the assumption that voters dislike the disruptions to state government services produced by delayed budgets. Everything else equal, voters will therefore prefer an on-time budget to a delayed one.

If voters dislike delays in the budget process, such delays should make reelection less likely for the politicians that voters perceive as responsible for them. This argument is closely related to the key assumptions underlying *procedural cartel theory* [18, 19] that 1) a party's reputation depends significantly on its record of legislative accomplishment, and 2) that this reputation in turn affects the probability of successful election outcomes for party members; indeed, Cox and McCubbins ([19], p. 22) use the decline in popularity of congressional Republicans following the 1995–96 federal budget battle as the main motivating example to support the former of these assumptions. The latter assumption is supported by a number of empirical studies showing that party brand favorability significantly affects outcomes of presidential, congressional and state legislative elections [17, 23, 24].

The key question separating our three hypotheses is *who* voters hold accountable for budget gridlock. Cox and McCubbins [18, 19] argue that legislative outcomes affect the record of legislative accomplishment for the majority party in the legislature only, which implies that majority party members are held accountable for legislative outcomes in state legislative elections, whereas minority party members are not; this can be seen as a variant of the responsible party government model [25, 26] in which the majority party in the legislature acts as the responsible party and voters should reward or punish members of this party for the performance of the government. In principle, supermajority requirements to pass the budget on time could present a problem for this argument, as they allow minority party legislators to obstruct the budget adoption process. However, such requirements are present in just three states [27], of which only one (Rhode Island) enters our final analysis sample.

The majority-party-as-responsible-party view has received support at the federal level, where empirical evidence shows that voters’ attitudes toward Congress’s job performance affect their support for candidates from the congressional majority party [28]. Opinion polls from federal government shutdown episodes are also mostly consistent with this view: respondents in such polls mostly blamed the Republican majorities in Congress during the federal shutdowns of 1995–96, 2013 and 2018 [29–31], whereas more respondents blamed President Trump than members of Congress during the 2018–19 shutdown [32].

It is also possible, however, that control over the legislative branch is not what really matters, as the executive branch is also deeply involved in the budget process [33, 34]: the governor submits an executive budget proposal to the legislature and also holds considerable veto power of the final proposal in many states. It is therefore possible that voters hold the governor accountable when the budget adoption process is gridlocked. Further, the literature on congressional politics has found that voters’ perceptions of the president’s performance have a significant impact on their attitudes towards members of the president’s party [35]. If a similar mechanism is present at the state level, we should expect voters to punish members of the governor’s party for budget gridlock, irrespective of who controls the state legislature.

Finally, a third possibility is that party affiliation is unimportant. Some researchers (e.g. [36]) have argued that political parties play no major role in explaining legislative gridlock in the context of the U.S. Congress. If voters in state legislative elections share this perception, all incumbent legislators may face adverse electoral consequences for budget impasses, and there is no reason to expect differential effects across party lines.

We summarize these ideas in the following three hypotheses:

**H1**: Voters disapprove of budget delays and punish members of the party that holds a majority in the legislature when they occur.
**H2**: Voters disapprove of budget delays and punish members of the governor’s party when they occur.
**H3**: Voters disapprove of budget delays and punish incumbent legislators from all parties when they occur.

These hypotheses lead to different predictions about the electoral outcomes for individual legislators. Under H1, budget delays should lower reelection rates for majority party incumbents, whereas minority party incumbents should experience no such effect. Moreover, if the damage to the majority party’s reputation affects *all* of its members, including non-incumbents, budget delays could even raise reelection rates for minority party incumbents. H2 predicts that budget delays lower reelection rates for incumbents from the *governor’s* party, while the effect should be zero or even positive for incumbents belonging to the opposite party. Finally, H3 predicts lower reelection probabilities for all incumbents in the state legislature, irrespective of party affiliation.

The impact of late budgets on the aggregate electoral performance of political parties under each of these hypotheses is not a priori clear, since it depends entirely on whether the ousted incumbents are replaced by someone from their own party (through primary election defeats), or by someone from another party. Obtaining precise predictions therefore requires further assumptions: under H1 and H2, voters assign responsibility for budgetary gridlock on a strictly partisan basis, so it seems natural to expect defeated incumbents to be replaced by someone from another party. H1 then predicts a negative effect of budget delays on the seat share obtained by the party that held a majority before the election, while H2 predicts a negative impact on the seat share for the governor’s party. Things are less clear under H3: since this hypothesis leaves no role for party affiliation, it is not clear who should be expected to replace the defeated incumbents. If these are mainly replaced by candidates from their own party, budget delays should have little or no impact on the party composition of the state legislature. In contrast, if they are replaced by someone from another party, we should expect the majority party to lose seats after a budget delay, since the majority party (by definition) has more incumbent members to start with. Even if incumbents are punished equally hard irrespective of their party affiliation, the total effect of anti-incumbent sentiment on party seat shares will work to the majority party’s disadvantage.

### Testing the hypotheses: The need for individual-level data

The discussion above illustrates that one cannot hope to learn much about the electoral consequences for individual state legislators by studying outcomes at the party level, since almost any observation at this level is consistent with at least two mutually exclusive hypotheses about individual-level effects. For example, suppose we observe a negative correlation between budget delays and the seat share obtained by the majority party in subsequent elections: this is consistent with H1, but also with H3 under the additional assumption that voters punish incumbents by electing someone from the opposite party. Similarly, observing a zero correlation between budget delays and subsequent electoral outcomes at the party level would be consistent with any of the hypotheses presented above if voters replace badly performing incumbents with other candidates from the same party, but it would also be consistent with the hypothesis that delays have no consequences for *any* incumbents. As these examples illustrate, we need data on electoral outcomes at the level of the individual legislator to properly test the three hypotheses above.

Further, while a party-level analysis cannot inform us about individual-level effects, the reverse is not true: exploiting the fact that we can separate incumbent defeats to same-party challengers from defeats to opposite-party challengers, we use our estimates to back out an estimated effect of budget delays on the party composition of the state legislature. However, we also supplement our main analysis for individual state legislators with a party-level analysis. This analysis provides a more direct estimate of the aggregate effect of budget gridlock, thus serving as a convenient robustness check of the results from our main analysis.

## Data and empirical specification

The objective of our empirical analysis is to examine the impact of a state’s history of late budgets since the previous election on the subsequent electoral performance of individual incumbents in the lower house of the state legislature. The unit of analysis is an individual incumbent legislator in a given state in a given year. The data source for electoral outcomes is the ICPSR data set on state legislative returns [37]. This data set contains information on more than 300,000 candidates who ran for state legislative office since 1967. For each lower-house election between 1989 and 2007, we identify all major-party incumbent legislators in the data and determine whether they ran for reelection, and if so, whether they succeeded. For incumbents who were not reelected, including those who did not run again, we also extract information about the party affiliation of their successor. As explained above, this is crucial for making inference about party-level consequences. However, it also forces us to limit our attention to electoral races in single-member districts, since there is no way of meaningfully identifying a unique successor in a multi-member district. Consequently, we exclude all incumbents running in multi-member districts from our main analysis, as well as those for which we lack information on party affiliation.

Finally, we use the ICPSR data to determine whether the incumbent was ineligible for reelection because of a binding term limit, and whether the incumbent ran for the state senate instead of the lower house. Information about term limits in state legislatures is from the NCSL. We limit our sample to incumbents that were in fact eligible for reelection, i.e. those that were neither term limited nor running for the state senate. For determining whether an incumbent belongs to the majority party in the legislature and/or to the same party as the governor, we rely on data provided by Carl Klarner [37].

Our measure of budgetary gridlock is based on data for late state budgets. The definition of a delay consists of two things: 1) the criteria for the budget process to be considered completed, and 2) the definition of the appropriate deadline by which this completion is supposed to be achieved. In US state governments the legislature and the governor often face different deadlines. Since we focus on legislatures, we define legislative budget gridlock as a situation in which the budget receives final legislative approval after the state legislature's deadline for passing the budget. Final legislative approval is achieved when the budget is passed in its final form by both chambers of the legislature. The deadline for achieving this varies from state to state: in some states, it coincides with the end of the fiscal year, while other states have earlier deadlines for the state legislature to pass the budget. For example, many state legislatures are required by constitution or statute to end their regular session by a certain date, and such requirements effectively constitute a deadline for all legislative activity, including passage of the budget.

The data for the legislatures’ budget enactment dates [38, 39] were collected from state legislatures' websites, archived newspaper articles and a survey sent to state budget officers. The survey asked state budget officers both to confirm the data collected from other sources and to provide information on budget passage dates not found through such sources. We refer to Andersen et al. [38, 39] for further details. Table A1 in S1 Online appendix provides an overview of the original sources of information on budget adoption dates, as well as the number of occurrences of budget gridlock in each state.

The budget enactment data covers the period 1988–2007, during which we observe 190 cases where the budget received final legislative passage after the legislature's state-specific deadline. This amounts to 26 percent of the budgets that we have data on. We let our analysis period begin in 1989 (the first election year with full data coverage for the preceding electoral cycle) and focus on the 33 states that experienced at least one budget delay between 1988 and 2007. These states held a combined total of 283 lower-house elections in the years 1989–2007. We exclude 41 of these from the analysis due to missing data on budget passage dates or election outcomes, or because all incumbents were elected in multi-member districts; for New Jersey and North Dakota, all elections held in this period fall under the latter category, so we exclude these states entirely. This leaves us with an analysis sample of 24,187 incumbent-year observations, distributed across 31 states and 242 lower-house elections in the years 1989 to 2007.

### Empirical specification

For each incumbent-year observation, we distinguish between three possible electoral outcomes: (1) Reelection, (2) being replaced by a candidate from the same party, and (3) being replaced by a candidate from another party. S1 Online appendix contains a more detailed definition. We estimate the probability of each of these outcomes using a multinomial logit model. As a robustness check, we also estimate linear regression models using the incumbent vote share as the dependent variable; see robustness section for further details.

The set of explanatory variables in the model includes individual-specific characteristics as well as state-level variables common to all incumbents running for reelection in state *s* in year *t*. The key explanatory variable measuring budget gridlock is the number of budget delays–as defined above–since the previous election in state *s*, normalized by the total number of budgets enacted in this time period. The normalization ensures comparability between states with two-year vs. four-year electoral cycles, and between states with annual vs. biennial budget enactment.

Motivated by the three hypotheses derived above, we allow for heterogeneous effects of the budget gridlock variable by interacting it with dummies indicating i) whether the incumbent belongs to the party that held a majority in the lower house of the state legislature before the election (from now on simply referred to as “the majority party”), and ii) whether the incumbent belongs to the same party as the governor (see below for analysis that include partisan balance in the senate). We analyze the two interactions in separate models. A specification allowing for a full three-way interaction is presented in S1 Online appendix.

The control variables include two measures of the incumbent’s previous electoral record (known as measures of *safety* in the literature): first, we include the vote share that (s)he obtained in the previous election. Second, we include the number of electoral races the incumbent has previously participated in, and its square, to proxy for campaigning experience.

The control variables also capture various other dimensions of the political setting facing the incumbent: previous studies have found state election outcomes to be affected by concurrent elections for higher office [9, 12]. To address this, we include the major-party vote share obtained by the gubernatorial candidate that belongs to the same party as the incumbent *j* in same-year gubernatorial races, as well as a dummy for gubernatorial election year. Similarly, we include the state-specific vote share obtained by the majority party's presidential candidate in same-year presidential elections. Voters in state elections may also be affected by events on the national scene in non-election years. To proxy for national shifts in party support, we construct a job approval rating index for the president at the time of the state election. The index measures the percentage of respondents in nation-wide Gallup polls who approve of the president's performance, minus the percentage that disapproves. We interact this variable with a dummy variable that takes the value 1 if incumbent *j* belongs to the same party as the president in year *t*. This dummy variable is also included directly with no interactions.

A third group of control variables captures the effects of fluctuations in the state economy that, in particular in times of crisis, can make it harder to reach a budget agreement [38]. These include the change in the state unemployment rate and the growth in real house prices since the previous year, the change in the ratio of state government expenditures to GDP since the previous election, the state government budget surplus in the year of the election, and tax increases enacted since the previous election, measured in percent of total general fund revenue. To allow for the possibility that these economic variables have different electoral consequences for different legislators, we include interactions between them and the dummy variables indicating membership of the majority party or of the governor’s party. The data sources for these and all other control variables are described in the S1 Online appendix.

Finally, state- and time fixed effects in incumbency advantage are allowed for by including full sets of state- and time dummies. The inclusion of state fixed effects is crucial, since it effectively controls for observed and unobserved time-invariant state characteristics that may correlate with both incumbent reelection rates and the frequency of budget delays. However, such characteristics could also have a differential impact on incumbents depending on their party affiliation. For example, one could imagine that majority party incumbents do worse than minority party incumbents in states with procedural rules that grant minority party members significant influence on legislative outcomes, such as supermajority requirement rules. If budget delays are also more common in such states, we might falsely interpret the weak performance of majority party incumbents relative to minority party incumbents in these states as a causal effect of budget gridlock. To account for this, we interact the state fixed effects with dummy variables for the incumbent’s party affiliation. Identification then comes from within-state comparisons between incumbents with the same party status (e.g. all majority party members or all members of the same party as the governor), but running in different years with a different record of legislative budget delays.

## Main results

### Descriptive statistics

Table 1 shows summary statistics for the observations in our sample, splitting them by whether at least one budget was delayed since the previous lower-house election in the state. In half of the elections, representing 12,153 of the 24,187 individual-level observations in the sample, there was at least one instance of budget gridlock. Conditional on at least one delay, the average share of delayed budgets since the previous election is 82%. This shows that budget gridlock is typically a recurring phenomenon, rather than an isolated, one-time event.

**Table 1:** Descriptive statistics.

	No gridlock	Gridlock	All
	------------means-------------		
Share of delayed budgets since previous election	0.00	0.82	0.41
Share of incumbents re-elected	0.83	0.80	0.82
Share of incumbents replaced by candidate from same party	0.11	0.12	0.12
Share of incumbents replaced by candidate from other party	0.06	0.07	0.07
Share of incumbents belonging to majority party in the lower house	0.60	0.61	0.60
Governor and majority in the lower house belong to diff. parties	0.45	0.62	0.53
Democratic majority in lower house	0.65	0.72	0.69
One-year change in state unemployment rate (ppts.)	-0.10	-0.19	-0.15
One-year growth rate in state house prices (%)	0.97	1.31	1.14
Change in state gov. expend. / GDP since previous election (ppts.)	0.26	0.24	0.25
Tax increases enacted since previous election (percent of total general fund revenue)	1.21	1.95	1.58
State government budget surplus in year of election (% of state GDP)	0.20	0.10	0.15
Number of elections	121	121	242
Number of incumbent-year obs.	12,034	12,153	24,187

The table shows descriptive statistics for our sample, split by budget gridlock status. The column labeled “No gridlock” shows statistics for the cases in which there were no occurrences of budget gridlock since the previous state legislative election. The column labeled “Gridlock” shows statistics for cases with at least one such occurrence. The column “All” shows statistics across all observations in our sample.

Table 1 shows that reelection rates are on average lower when there is a budget delay (80%) than when all budgets are adopted on time (83%). Conversely, the share of incumbents that are replaced by someone from their own party is slightly higher in the former case (12% vs. 11%), and so is the share of incumbents replaced by someone from the opposite party (7% vs. 6%).

Budget gridlock correlates strongly with divided government: 62 percent of the elections characterized by gridlock took place when the governor and the majority in the lower house of the legislature belonged to different parties, against only 45 percent of the delay-free elections. Things are less clear for the economic variables: the average one-year change in the state unemployment rate is a drop of .19 percentage points for elections following budget gridlock, compared to .10 percentage points for delay-free elections. Similarly, the election years following gridlocked cycles are characterized by stronger house price increases than those following cycles with timely budget adoption. In contrast, the average state government budget balance is weaker when there has been at least one delay, and taxes are raised more in such years, suggesting that gridlock occurs more frequently when the state government is under fiscal pressure; these observations are consistent with findings in the existing literature that the probability of budgetary gridlock is higher under divided government [38, 40] and in times of changing fiscal circumstances, especially fiscal downturns [38].

### Majority party incumbents vs. minority party incumbents

**Fig 1:**
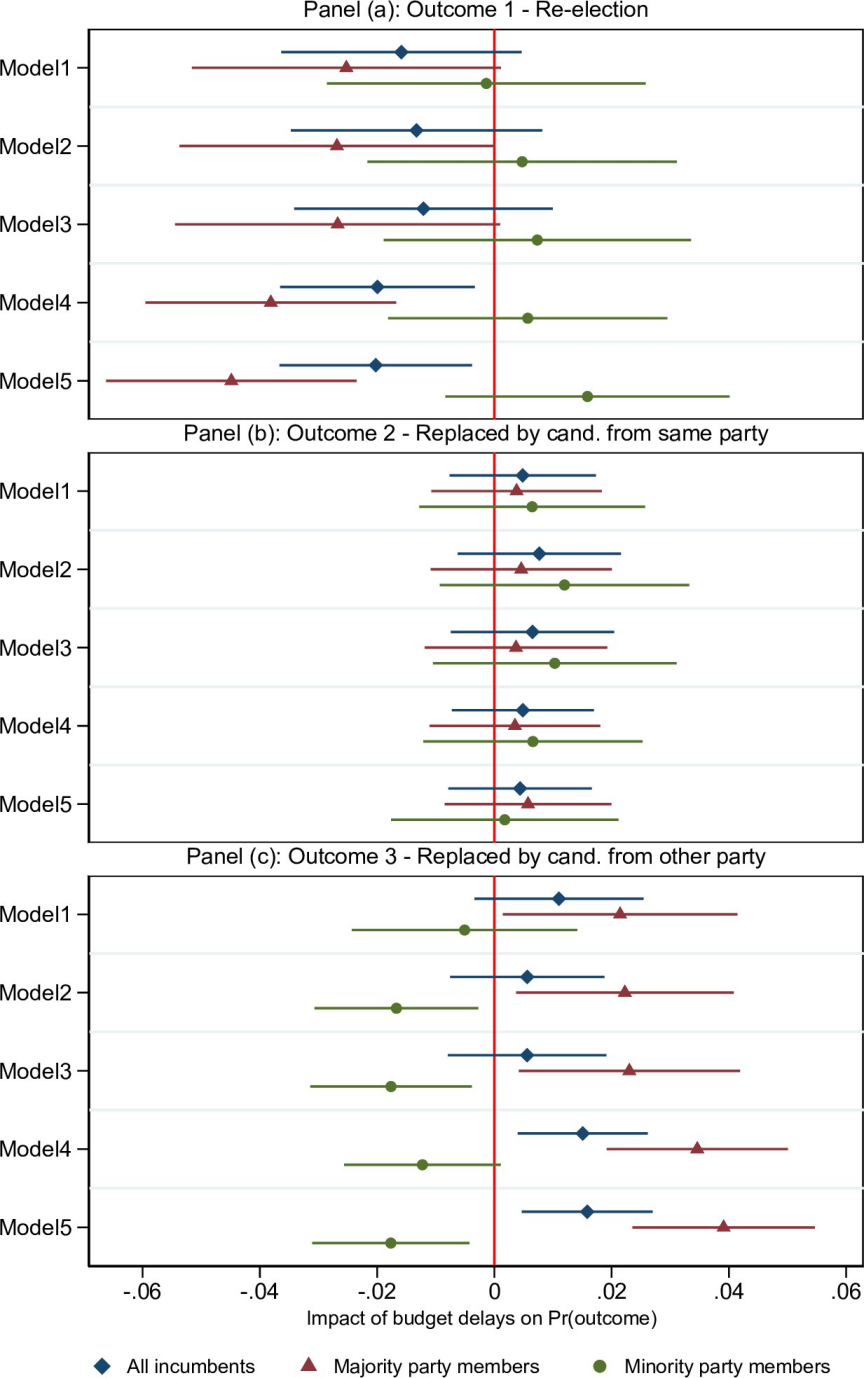
Effects of budget gridlock: majority vs. minority party members. The figure shows average marginal effects of changing the budget gridlock variable from zero to one on the probability of each of three possible election outcomes for incumbent legislators, allowing different effects for majority vs. minority party members. Panel (a) shows results for outcome 1 (re-election), while Panels (b) and (c) show results for outcomes 2 (replaced by same-party candidate) and 3 (replaced by other-party candidate), respectively. Each row corresponds to a different model specification: Model 1 includes only time dummies and dummies for majority party membership and membership of the governor’s party as controls. The following controls are added sequentially in the other models: electoral record and political controls (Model 2), economic controls x majority party membership (Model 3), state fixed effects (Model 4), and state fixed effects x majority party membership (Model 5). Horizontal bars around point estimates illustrate 95% confidence intervals. Standard errors are estimated allowing for clustering at the state-year level. All estimates and further details about each model specification are reported in Table A2 in S1 Online appendix.

We now turn to the results for the multinomial logit model described above. We begin with a version of the model in which the budget gridlock variable is interacted with a dummy for membership of the majority party in the lower house of the legislature. The results are shown in Fig 1 and presented in table format in S1 Online appendix, Table A2. Since the parameter estimates do not have straightforward interpretations, we show the average partial effects of budget delays on the predicted probabilities of each of the three electoral outcomes, calculated as average changes in probabilities when the budget gridlock variable changes from zero to one and all other explanatory variables are evaluated at their actual values. Panel (a) of Fig 1 reports average partial effects on the probability of reelection. The estimates within each row correspond to a particular version of the empirical model: Model 1 is a stripped-down version in which only time dummies and the dummy variables for majority party membership and membership of the governor’s party are included as controls. Models 2–5 sequentially add further controls and state fixed effects.

The blue diamond-shaped series shows the average partial effects on the probability of reelection for all incumbents, regardless of their partisan affiliation. The estimated effect is negative across all models, ranging from -1.2 to -2.0 percentage points, and is statistically significant in Models 4 and 5, which include state fixed effects. However, the figure also shows that these estimates conceal an important difference between majority party members and minority party members: for majority party incumbents, the estimated effect ranges from -2.5 to -4.5 percentage points and it is significant at the 1 percent level when state fixed effects are included. For minority party members, we find a positive estimate in four out of five columns, but it is small and never statistically significant. The difference between the two groups is statistically significant at the 5 percent level or less once economic controls are included in Model 3.

Panels (b) and (c) show the corresponding effects on the probabilities of being replaced by a candidate from the same party and from the opposite party, respectively. Beginning with the latter, Panel (c) shows the mirror image of that in Panel (a) for majority party incumbents: for this group, budget gridlock increases the probability of being replaced by a candidate from a different party by 2–4 percentage points. The results for minority party incumbents are again quite different: if anything, the results suggest that the probability of being replaced by someone from another party decreases for this group. At 1–2 percentage points, the estimates are moderate in size, but they are numerically larger than the opposite-signed estimates in Panel (a) and also more precisely estimated (p-value below .05 in three out of five models). The difference between majority party incumbents and minority party incumbents is again sizeable and strongly significant.

In contrast, Panel (b) shows no significant effect of budget gridlock on the probability of being replaced by a same-party candidate for either group. For minority party incumbents, this leaves us with some ambiguity about the consequences of gridlock: as shown in Panel (c), we find a (mostly) significant negative impact on the probability that these incumbents are replaced by majority party challengers. By definition, this must result from either an increase in the probability of reelection or a decrease in the probability of being replaced by someone from the same party (or some combination of the two); however, our analysis does not produce sufficiently precise estimates of these two effects to determine which is more relevant.

### Members of governor’s party vs. non-members

**Fig 2:**
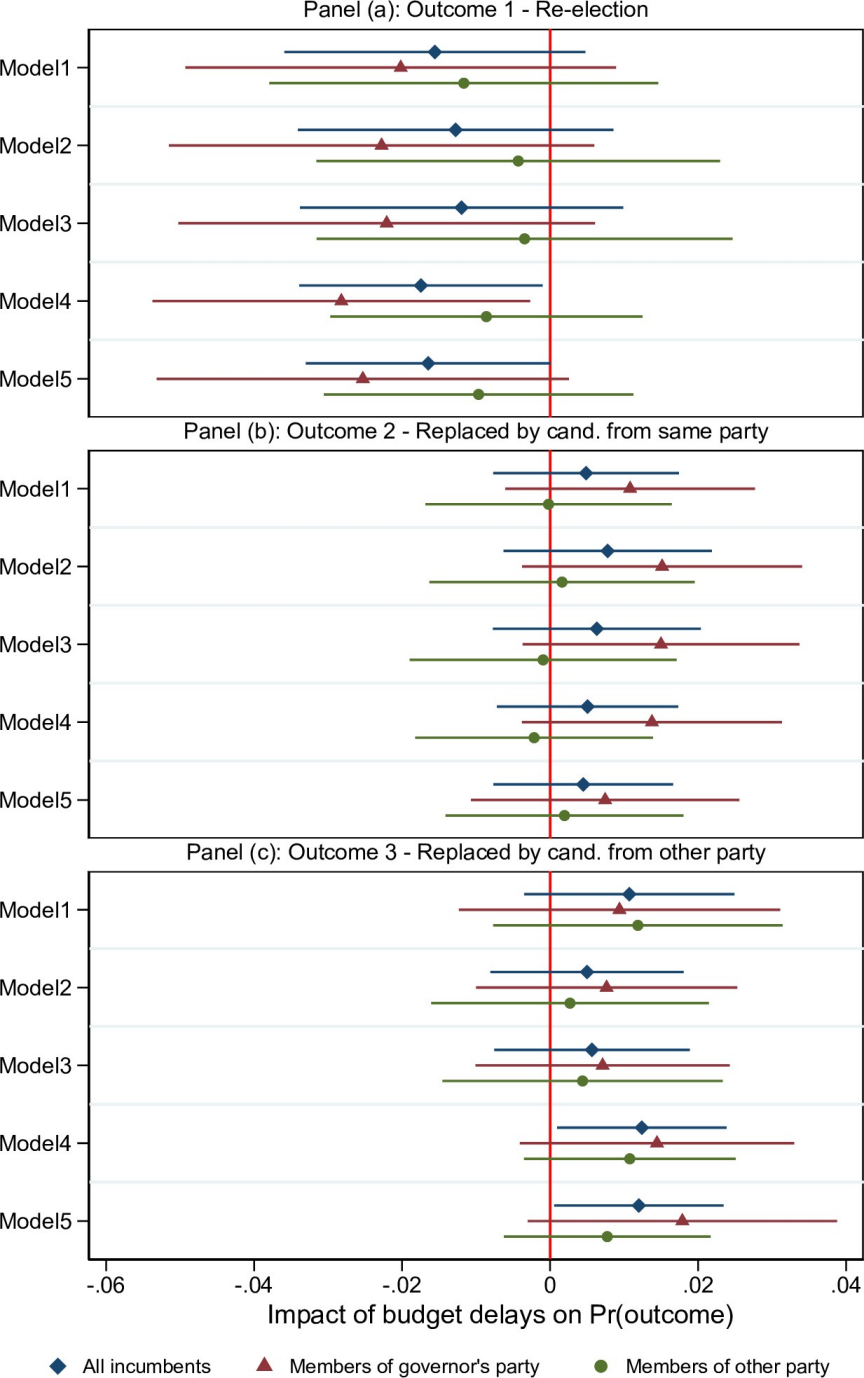
Effects of budget gridlock: members of governor’s party vs. non-members. The figure shows average marginal effects of changing the budget gridlock variable from zero to one on the probability of each of three possible election outcomes for incumbent legislators, allowing different effects for members of the governor’s party vs. non-members. Panel (a) shows results for outcome 1 (re-election), while Panels (b) and (c) show results for outcomes 2 (replaced by same-party candidate) and 3 (replaced by other-party candidate), respectively. Each row corresponds to a different model specification: Model 1 includes only time dummies and dummies for majority party membership and membership of the governor’s party as controls. The following controls are added sequentially in the other models: electoral record and political controls (Model 2), economic controls x majority party membership (Model 3), state fixed effects (Model 4), and state fixed effects x majority party membership (Model 5). Horizontal bars around point estimates illustrate 95% confidence intervals. Standard errors are estimated allowing for clustering at the state-year level. All estimates and further details about each model specification are reported in Table A3 in S1 Online appendix.

Hypothesis H2 predicts that rather than majority party membership, what matters for the effect of budget gridlock on an incumbent’s reelection prospects is whether (s)he belongs to the governor’s party. To test this, we estimate a version of the multinomial logit model in which an interaction term between the gridlock variable and membership of the governor’s party is included. The results are shown in Fig 2, which parallels Fig 1 except that the central distinction is now between members of the governor’s party vs. non-members, rather than majority vs. minority party members. Table A3 in S1 Online Appendix provides further details about the estimates shown in the figure.

As in Fig 1, Panel (a) of Fig 2 shows a negative effect of budget gridlock on the probability of reelection in the order of 1–2 percentage points when we average across all incumbents. However, the results do not reveal any significant difference between incumbents when split by whether they belong to the same party as the governor: the estimated effect of budget gridlock is numerically somewhat larger for members of the governor’s party than for non-members, but the difference between the two groups is never significant. Similarly, we see no solid evidence of a differential impact of budget gridlock between these two groups of incumbents in panels (b) and (c) of the table, which show the estimated effects on the probabilities of being replaced by another candidate from the same party or the opposite party.

### A full three-way interaction

To complete the analysis, we have also estimated a version of the model that includes a full three-way interaction between budget gridlock, majority party membership, and membership of the governor’s party. The results are reported in Table A4 in S1 Online appendix. In short, allowing for a full three-way interaction does not change the main conclusions drawn above: the difference between majority and minority party incumbents is sizeable and statistically significant at the five percent level, regardless of which party controls the executive branch of the state government. In contrast, we find no significant effects of the governor’s party affiliation, neither for majority party incumbents nor minority party incumbents. Our results therefore strongly support the view that voters hold members of the majority party in the legislature accountable for budget gridlock, as predicted by H1.

### Shutdown provisions and accountability

The consequences of not passing the budget on time vary considerably across states [38]. In some states, continuing resolutions keep the government running at normal or close to normal scale. In other states, government employees are sent home without pay and all non-essential facilities are closed. We now explore how this institutional variation interacts with voter reactions to late state budgets.

Table 2 shows that the effects of budget gridlock on reelection probabilities are different in states that have no provisions in place that prevent the state government from shutting down following a delayed budget (26 percent of cases), compared to states where such provisions exist (74 percent of cases). Consistent with our earlier results, minority party members are not affected by budget delays in either case.

**Table 2:** Provisions to keep government operations running.

	(1)		(2)
	No provisions		Provisions
	Outcome 1: Reelection		
Majority party members	-0.061		-0.034
	(0.019)		(0.013)
Minority party members	0.001		0.014
	(0.018)		(0.016)
Difference	-0.063		-0.049
	(0.027)		(0.018)
No. of states	9		22
No. of elections	63		179
Observations	7,029		17,150

The table reports average marginal effects of changing the budget gridlock variable from zero to one on the probability of reelection for incumbent legislators, based on estimation of a multinomial logit model with three possible outcomes. Column (1) reports results for the subsample of states in which there are no provisions to prevent the government from shutting down in the event of a delayed budget. Column (2) reports results for the subsample of states that do have such provisions. Control variables corresponding to those in Model 4 of Fig 1 included in all columns. Standard errors (in parentheses) are estimated allowing for clustering at the state-year level.

In contrast, we find that majority party incumbents’ probability of reelection decreases by 6.1 percentage points in states where a late budget leads to a government shutdown, compared to 3.4 percentage points in states with no-shutdown provisions. This suggests that the electoral response is stronger when the consequences of gridlock in terms of welfare are more severe, consistent with standard accounts of economic voting [41].

### Open-seat races

The result that budget gridlock lowers the probability that minority party incumbents lose their seats to candidates from other parties suggests that the adverse effects for majority party candidates are not confined to incumbent legislators: even non-incumbent challengers from this party perform worse when state budgets are delayed. This is consistent with findings showing that voters’ perception of a party’s brand affects the electoral prospects of any candidate running under that party label [17, 23, 42].

Table 3 provides further evidence in support of this idea: here we report estimates for the subsample of open-seat races, i.e. elections in which the incumbent did not run for reelection. These races are interesting because they involve only non-incumbent candidates, so any systematic effect of budget gridlock suggests that voters’ attitudes towards the party brand are affected. Since reelection is by definition ruled out, we now use a binary indicator for outcome 3 (succeeded by someone from another party) as the dependent variable and estimate the model with logistic regression.

**Table 3:** Open-seat races.

	Outcome 3: Replaced by candidate from other party	

Open-seat races where incumbent	0.091	
belongs to majority party	(0.025)	
Open-seat races where incumbent	-0.022	
belongs to minority party	(0.025)	
No. of states	31	
No. of elections	240	
Observations	3,460	

The table reports average marginal effects of changing the budget gridlock variable from zero to one on the probability that the incumbent is replaced by someone from another party. Only lower-house elections in which the incumbent legislator does not run are included. The control variables are the same as in Model 4 of Fig 1. Standard errors (in parentheses) are estimated allowing for clustering at the state-year level.

If budget gridlock hurts non-incumbent majority party candidates, we should expect a positive sign on the delay variable in races where the outgoing incumbent belongs to the majority party, and a negative sign when the incumbent belongs to the minority party. As shown in Table 3, this is indeed what we find: in districts with a majority party incumbent not running for reelection, a unit increase in the budget gridlock variable is associated with a 9 percentage points increase in the probability of electing someone from the opposite party. In open-seat races with minority party incumbents the estimated effect is negative, as expected, but numerically much smaller and statistically insignificant. In sum, these results suggest that majority party candidates are hurt by budget gridlock also in open seat races, but only significantly so when the outgoing incumbent is also a member of the majority party. This is consistent with the idea of party labels or brands being important for voter decision-making.

## Robustness

We have performed a number of robustness tests of our main results. This section summarizes the main points; full results are reported in S1 Online appendix. First, our main result–the difference in electoral consequences of budget gridlock between majority vs. minority party incumbents–is robust to alternative definitions of the budget gridlock variable, including whether the state budget was delayed in *any* year since the previous election, or in the *same* year as the current election. We also show that our main results are qualitatively unchanged if we use a simple binary logit model for reelection vs. not reelection, or linear models with individual incumbent vote shares as the dependent variable.

The main analysis includes open-seat races in the estimation sample, treating cases in which the incumbent does not run for reelection as incumbent defeats. As explained in the discussion above, the negative effect of budget gridlock on majority party candidates’ electoral prospects is strongly present in these races. At the same time, the robustness tests show that our results also hold if we instead exclude open-seat races and focus exclusively on races where the incumbent did in fact run for reelection. This means that our results are not driven by incumbents choosing not to run for reelection, contrasting with Binder’s [4] observation for the US Congress that there is no effect of (her measure of) gridlock on the probability of reelection conditional on seeking such reelection; she instead identifies a small negative effect of gridlock on the probability of seeking reelection, and concludes that gridlock makes politicians leave politics.

Throughout the paper, we have equated partisan control over the state legislature with control over the lower house, ignoring the party composition in state senates. One can argue that split party control over the two chambers of the legislature blurs who is responsible for legislative outcomes, so it is possible that voters respond differently in lower house elections depending on which party controls the state senate. To check whether our results are affected by any such heterogeneity in voter responses, we have estimated our model on the subsample of observations stemming from elections in which the same party controlled both chambers. The results are virtually identical to those in the baseline analysis. If we instead limit the sample to elections following split-legislature cycles, standard errors increase somewhat due to a much smaller sample size, but the point estimates of the marginal effects are very similar to the baseline estimates.

Another potential concern is that our budget gridlock measure captures the state government being under fiscal pressure, despite our efforts to control for state economic and fiscal indicators known to correlate with late budgets [38]. To explore this, we have estimated our baseline model on the subsample of elections for which the state unemployment rate fell over the 12 months leading up to the election, indicating a strong state economy. Here, too, we find a strong negative impact of budget gridlock on the reelection prospects for majority party incumbents, whereas there is no significant effect for minority party incumbents. This supports the interpretation that gridlock in itself causes voters to punish majority party incumbents.

Finally, the data on budget enactment dates used to construct the gridlock variable has missing observations in some states, especially in the early years of our data set. As a final robustness check, we have therefore estimated our model on the 20 states in our sample for which we have information about budget enactment dates in all years in the analysis period. The results are again very similar to those in the baseline estimation.

## Party composition in state legislatures

We can use the estimates presented above to back out an estimate of the effect of budget gridlock on the aggregate electoral performance of the majority party in the state legislature. For example, a simple back-of-the-envelope calculation based on the estimates shown in Fig 1, Model 4 tells us that the average effect of changing the budget gridlock variable from zero to one on the majority party’s seat share is a drop of 2.6 percentage points, with a 95% confidence interval from 1.6 to 3.6 (see S1 Online appendix for details).

To obtain direct estimates of the effect of budget gridlock on party-level electoral outcomes, we have also run a series of regressions in which the seat share obtained by the majority party is regressed on our budget gridlock measure and a set of controls paralleling those included in the individual-level estimations. The unit of analysis is a lower-house election in a given state in a given year. In the interest of space, we confine the detailed description of these regressions to the S1 Online appendix and only summarize the key results: using the same sample of 242 elections as in our main analysis, we find that changing the gridlock variable from zero to one reduces the majority party’s vote share by 2.9 percentage points (significant at the 1 percent level). This is very close to the 2.6 percentage points estimate reported above. Extending the sample to include elections in which incumbents run in multi-member districts does not change the results in any significant way. Further, we find a budget gridlock effect on the majority party’s seat share of -3.4 percentage points when the governor also belongs to this party, versus -2.1 percentage points in the opposite case. Again, these point estimates are virtually identical to the ones from our main analysis of electoral outcomes for individual incumbents.

Are these reported effects on the majority party’s seat share small or large? In the order of 2–3 percentage points, the estimated effects of budget gridlock are not huge, but large enough to be relevant in a fairly large share of lower-house elections: of the 262 elections included in our analysis, 55 (or 21%) resulted in a seat share within 3 percentage points from the 50% threshold for the party that had a majority before the election. Combined with our estimates of the electoral effects of budget gridlock, this suggests that about one fifth of state legislative elections are sufficiently close that whether the state budget is passed on time or not could be decisive for which party will gain control over the state legislature. Another way to assess the magnitude of the gridlock effect is to compare it to the estimated effects for the control variables. In general, however, we find limited impact from state economic and fiscal performance on lower-house state legislative elections, consistent with previous findings [9, 12]. For example, the party-level analysis mentioned above suggests that a one percentage point increase in the one-year change in the state unemployment rate reduces the seat share obtained by the majority party by only 0.3 percentage points on average, and by 1.2 percentage point under unified government, and while both are imprecisely estimated, our estimates of the electoral impact of budget gridlock appear quite sizeable in comparison.

## Concluding remarks

Legislative gridlock is a matter of key concern in democratic politics, since it has the potential to hinder political action on important issues in a timely fashion. In this article, we study voter reactions to a particular set of gridlock instances, budget gridlock in US states, with the aim of understanding whether and how voters contribute to mitigating the problem of gridlock by holding elected lawmakers collectively accountable for legislative impasses.

We find strong evidence showing that voters in state legislative elections do react to occurrences of budget gridlock: on average, reelection rates for incumbent legislators drops by 1–2 percentage points. The effect is entirely driven by lower reelection rates for members of the majority party in the legislature. We find that these incumbents are always punished by voters for budget gridlock, no matter which party controls the other branches of the state government. Non-incumbent members of the majority party also suffer electorally and are less successful in defeating minority party incumbents when the state budget has been delayed. Although the effect is smaller than for incumbent majority party members, this is consistent with a key assertion in influential theories of party organization, namely that a poor legislative performance hurts the brand of the majority party in the legislature, to the detriment of *all* candidates running under that brand. For the majority party as a whole, we find that budget gridlock reduces the seat share obtained in the subsequent election by about 2–3 percentage points on average. For one fifth of the state legislative elections in our analysis sample, a change in the party composition of legislative seats of this magnitude would be enough to tip the partisan balance of power in the lower house of the state legislature from one party to the other. Our results therefore indicate that voters hold members of the majority party collectively accountable for budget gridlock to an extent that leaders of the party often cannot afford to ignore. In contrast, we find no evidence for the hypothesis that voters base their judgement of who is responsible for budget gridlock on the party affiliation of the governor and act accordingly when casting their ballot.

The focus in this paper is on *collective* accountability for budget gridlock: we explore whether different groups of incumbents–as defined by their party affiliation–experience different electoral consequences when they collectively fail to pass a state budget on time. A related question is whether voters hold incumbent legislators *individually* accountable for their roles in the budget adoption process. For example, one can imagine that legislators who have a direct responsibility for getting the budget passed–through membership of an appropriations committee or the party leadership, for example–are particularly harshly punished when the legislative process reaches a deadlock. On the other hand, one can also imagine that some legislators can actually benefit personally from obstructing the legislative process, even while their fellow party members suffer as a group, if by doing so they can convince voters of their “toughness.” [43]. Exploring this issue would require detailed and quantifiable data on each legislator’s formal responsibilities in the budget adoption process, as well on his or her personal conduct and actions during that process: we believe this to be an interesting path for future research.

## Supporting information

S1 Online appendix (DOCX)

S1 Data files (ZIP)
