# Supplementary material for: Irresponsible parties, responsible voters? Legislative gridlock and collective accountability
Source: PLoS One. 2020 Mar 2;15(3):e0229789. doi: 10.1371/journal.pone.0229789 (PMC7051087; doi:10.1371/journal.pone.0229789)
Supplement: S1 Online appendix — (DOCX) [file pone.0229789.s001.docx]

# S1 Online appendix.

# Variables and data sources

**Definition of outcome variable in multinomial logit models**

In all multinomial logit analyses, we use a categorical dependent variable with the following three outcomes:

1. Re-election
2. Replaced by a candidate from the same party
3. Replaced by a candidate from another party

Outcome (3) includes cases in which the incumbent is replaced by a non-major-party candidate. Cases where an eligible incumbent does not run for reelection (unless running for the state senate) are treated in the same way as incumbent defeats and coded as either (2) or (3), depending on the identity of the successor. Our reason for taking this approach is that an incumbent’s decision on whether or not to run may be highly strategic (Jacobson 1989). In the robustness section, we document that our main results are insensitive to limiting the sample to races in which the incumbent did in fact run for reelection.

**Data sources for control variables**

- **Incumbent vote share and number of previous campaigns:** Klarner, C.E, W.D. Berry, T. Carsey, M. Jewell, R. Niemi, L. Powell, and J. Snyder. 2013. “State Legislative Election Returns (1967-2010).” ICPSR34297-v1. Ann Arbor, MI: Inter-university Consortium for Political and Social Research. <http://doi.org/10.3886/ICPSR34297.v1>
- **Major-party vote share obtained by same-party gubernatorial candidate:** Statistical Abstract of the United States, various years.
- **State-level vote shares for presidential candidates**: uselectionatlas.org
- **Presidential approval ratings in Gallup polls:** <http://ropercenter.cornell.edu/>.
- **State unemployment rate**: Bureau of Labor Statistics
- **State house price growth**: Federeal Housing Agency
- **State government expenditure and budget surpluses**: US Census Bureau
- **State Gross Domestic Product**: Bureau of Economic Analysis
- **State tax increases**: The National Association of State Budget Officers: *The Fiscal Survey of States*

**Budget gridlock in each state: Occurrences and data sources**

# Main results

**Table A2. Effects of budget gridlock: majority vs. minority party incumbents**

|  | Model 1 |  | Model 2 |  | Model 3 |  | Model 4 |  | Model 5 |  |
| --- | --- | --- | --- | --- | --- | --- | --- | --- | --- | --- |
|  | Outcome 1: Re-election | | | | | | | | | |
|  |  |  |  |  |  |  |  |  |  |  |
| All incumbents | -0.016 |  | -0.013 |  | -0.012 |  | -0.020 |  | -0.020 |  |
|  | (0.010) |  | (0.011) |  | (0.007) |  | (0.008) |  | (0.010) |  |
| Majority party members | -0.025 |  | -0.027 |  | -0.027 |  | -0.038 |  | -0.045 |  |
|  | (0.013) |  | (0.014) |  | (0.014) |  | (0.011) |  | (0.011) |  |
| Minority party members | -0.001 |  | 0.005 |  | 0.007 |  | 0.006 |  | 0.016 |  |
|  | (0.014) |  | (0.013) |  | (0.013) |  | (0.012) |  | (0.012) |  |
| Difference | -0.024 |  | -0.032 |  | -0.034 |  | -0.044 |  | -0.061 |  |
|  | (0.018) |  | (0.016) |  | (0.016) |  | (0.016) |  | (0.016) |  |
|  | Outcome 2: Replaced by candidate from same party | | | | | | | | | |
|  |  |  |  |  |  |  |  |  |  |  |
| All incumbents | 0.005 |  | 0.008 |  | 0.007 |  | 0.005 |  | 0.004 |  |
|  | (0.006) |  | (0.007) |  | (0.007) |  | (0.006) |  | (0.006) |  |
| Majority party members | 0.004 |  | 0.005 |  | 0.004 |  | 0.004 |  | 0.006 |  |
|  | (0.007) |  | (0.008) |  | (0.008) |  | (0.007) |  | (0.007) |  |
| Minority party members | 0.006 |  | 0.012 |  | 0.010 |  | 0.007 |  | 0.002 |  |
|  | (0.010) |  | (0.011) |  | (0.011) |  | (0.010) |  | (0.010) |  |
| Difference | -0.003 |  | -0.007 |  | -0.007 |  | -0.003 |  | 0.004 |  |
|  | (0.011) |  | (0.012) |  | (0.012) |  | (0.011) |  | (0.011) |  |
|  | Outcome 3: Replaced by candidate from other party | | | | | | | | | |
|  |  |  |  |  |  |  |  |  |  |  |
| All incumbents | 0.011 |  | 0.006 |  | 0.006 |  | 0.015 |  | 0.016 |  |
|  | (0.007) |  | (0.004) |  | (0.007) |  | (0.006) |  | (0.006) |  |
| Majority party members | 0.021 |  | 0.022 |  | 0.023 |  | 0.035 |  | 0.039 |  |
|  | (0.010) |  | (0.009) |  | (0.010) |  | (0.008) |  | (0.008) |  |
| Minority party members | -0.005 |  | -0.017 |  | -0.018 |  | -0.012 |  | -0.018 |  |
|  | (0.010) |  | (0.007) |  | (0.007) |  | (0.007) |  | (0.007) |  |
| Difference | 0.027 |  | 0.039 |  | 0.041 |  | 0.047 |  | 0.057 |  |
|  | (0.014) |  | (0.011) |  | (0.010) |  | (0.010) |  | (0.010) |  |
| Party affiliation dummies | Yes |  | Yes |  | Yes |  | Yes |  | Yes |  |
| Electoral record controls | No |  | Yes |  | Yes |  | Yes |  | Yes |  |
| Political controls | No |  | Yes |  | Yes |  | Yes |  | Yes |  |
| Economic controls | No |  | No |  | Yes |  | Yes |  | Yes |  |
| Economic controls x majority party | No |  | No |  | Yes |  | Yes |  | Yes |  |
| State fixed effects | No |  | No |  | No |  | Yes |  | Yes |  |
| State fixed effects x majority party | No |  | No |  | No |  | No |  | Yes |  |
| Year fixed effects | Yes |  | Yes |  | Yes |  | Yes |  | Yes |  |
| No. of states | 31 |  | 31 |  | 31 |  | 31 |  | 31 |  |
| No. of elections | 242 |  | 242 |  | 242 |  | 242 |  | 242 |  |
| Observations | 24,187 |  | 24,179 |  | 24,179 |  | 24,179 |  | 24,179 |  |

The table reports average marginal effects of changing the budget gridlock variable from zero to one on the probability of each election outcome for incumbent legislators. Each column shows results for a different version of the empirical model, corresponding to those shown in Fig. 1. Each of the first three rows within each panel reports the estimated effect on the probability of a particular outcome for a particular type of incumbent. The rows labeled "Difference" report the differences in effects between the two rows immediately above. Standard errors (in parentheses) are estimated allowing for clustering at the state-year level.

**Table A3. Effects of budget gridlock: members of governor’s party vs. non-members**

|  | Model 1 |  | Model 2 |  | Model 3 |  | Model 4 |  | Model 5 |  |
| --- | --- | --- | --- | --- | --- | --- | --- | --- | --- | --- |
|  | Outcome 1: Re-election | | | | | | | | | |
|  |  |  |  |  |  |  |  |  |  |  |
| All incumbents | -0.016 |  | -0.013 |  | -0.012 |  | -0.017 |  | -0.016 |  |
|  | (0.010) |  | (0.011) |  | (0.011) |  | (0.008) |  | (0.008) |  |
| Members of governor's party | -0.020 |  | -0.023 |  | -0.022 |  | -0.028 |  | -0.025 |  |
|  | (0.015) |  | (0.015) |  | (0.014) |  | (0.013) |  | (0.014) |  |
| Non-members of governor's party | -0.012 |  | -0.004 |  | -0.003 |  | -0.009 |  | -0.010 |  |
|  | (0.013) |  | (0.014) |  | (0.014) |  | (0.011) |  | (0.011) |  |
| Difference | -0.009 |  | -0.018 |  | -0.019 |  | -0.020 |  | -0.016 |  |
|  | (0.019) |  | (0.019) |  | (0.018) |  | (0.017) |  | (0.018) |  |
|  | Outcome 2: Replaced by candidate from same party | | | | | | | | | |
|  |  |  |  |  |  |  |  |  |  |  |
| All incumbents | 0.005 |  | 0.008 |  | 0.006 |  | 0.005 |  | 0.004 |  |
|  | (0.006) |  | (0.007) |  | (0.007) |  | (0.006) |  | (0.006) |  |
| Members of governor's party | 0.011 |  | 0.015 |  | 0.015 |  | 0.014 |  | 0.007 |  |
|  | (0.009) |  | (0.010) |  | (0.010) |  | (0.009) |  | (0.009) |  |
| Non-members of governor's party | 0.000 |  | 0.002 |  | -0.001 |  | -0.002 |  | 0.002 |  |
|  | (0.008) |  | (0.009) |  | (0.009) |  | (0.008) |  | (0.008) |  |
| Difference | 0.011 |  | 0.014 |  | 0.016 |  | 0.016 |  | 0.005 |  |
|  | (0.011) |  | (0.012) |  | (0.012) |  | (0.012) |  | (0.012) |  |
|  | Outcome 3: Replaced by candidate from other party | | | | | | | | | |
|  |  |  |  |  |  |  |  |  |  |  |
| All incumbents | 0.011 |  | 0.005 |  | 0.006 |  | 0.012 |  | 0.012 |  |
|  | (0.007) |  | (0.007) |  | (0.007) |  | (0.006) |  | (0.006) |  |
| Members of governor's party | 0.009 |  | 0.008 |  | 0.007 |  | 0.014 |  | 0.018 |  |
|  | (0.011) |  | (0.009) |  | (0.009) |  | (0.009) |  | (0.011) |  |
| Non-members of governor's party | 0.012 |  | 0.003 |  | 0.004 |  | 0.011 |  | 0.008 |  |
|  | (0.010) |  | (0.010) |  | (0.010) |  | (0.007) |  | (0.007) |  |
| Difference | -0.002 |  | 0.0049 |  | 0.0027 |  | 0.0037 |  | 0.0102 |  |
|  | (0.015) |  | (0.013) |  | (0.013) |  | (0.012) |  | (0.013) |  |
| Party affiliation dummies | Yes |  | Yes |  | Yes |  | Yes |  | Yes |  |
| Electoral record controls | No |  | Yes |  | Yes |  | Yes |  | Yes |  |
| Political controls | No |  | Yes |  | Yes |  | Yes |  | Yes |  |
| Economic controls | No |  | No |  | Yes |  | Yes |  | Yes |  |
| Economic controls x governor's party | No |  | No |  | Yes |  | Yes |  | Yes |  |
| State fixed effects | No |  | No |  | No |  | Yes |  | Yes |  |
| State fixed effects x governor's party | No |  | No |  | No |  | No |  | Yes |  |
| Year fixed effects | Yes |  | Yes |  | Yes |  | Yes |  | Yes |  |
| No. of states | 31 |  | 31 |  | 31 |  | 31 |  | 31 |  |
| No. of elections | 242 |  | 242 |  | 242 |  | 242 |  | 242 |  |
| Observations | 24,187 |  | 24,179 |  | 24,179 |  | 24,179 |  | 24,179 |  |

The table reports average marginal effects of changing the budget gridlock variable from zero to one on the probability of each election outcome for incumbent legislators. Each column shows results for a different version of the empirical model, corresponding to those shown in Fig. 2. Each of the first three rows within each panel reports the estimated effect on the probability of a particular outcome for a particular type of incumbent. The rows labeled "Difference" report the differences in effects between the two rows immediately above. Standard errors (in parentheses) are estimated allowing for clustering at the state-year level.

The analysis in the main text presents results for regressions in which the budget gridlock variable is interacted with *either* a dummy for majority party membership *or* a dummy for membership of the governor’s party. Table A4 reports results from a version of the multinomial logit model that includes a full three-way interaction term between these three variables. . We report the average partial effects of budget gridlock on the probability of outcome 1, reelection. Column (1) reports results from a specification paralleling Model 4 of Fig. 1 in the main text: All political and economic controls are included, but the economic controls are now, like the gridlock variable, included in a full three-way interaction with the dummies for majority party membership and membership of the governor’s party. State fixed effects are included, but not interacted with either of the two-party affiliation dummies. Such two-way interactions are included in column (2), while column (3) includes a full three-way interaction between the state fixed effects and the party affiliation dummies.^[[1]](#footnote-1)^

**Table A4: Three-way interaction btw. gridlock, majority party, and governor’s party**

|  | (1) |  | (2) |  | (3) |  |
| --- | --- | --- | --- | --- | --- | --- |
|  | Outcome 1: Re-election | | | | | |
|  |  |  |  |  |  |  |
| Majority party members, governor from same party | -0.051 |  | -0.060 |  | -0.069 |  |
|  | (0.018) |  | (0.019) |  | (0.021) |  |
| Majority party members, governor from different party | -0.025 |  | -0.039 |  | -0.044 |  |
|  | (0.014) |  | (0.014) |  | (0.015) |  |
| Difference | -0.025 |  | -0.021 |  | -0.025 |  |
|  | (0.022) |  | (0.023) |  | (0.027) |  |
| Minority party members, governor from same party | -0.004 |  | 0.012 |  | 0.004 |  |
|  | (0.017) |  | (0.018) |  | (0.019) |  |
| Minority party members, governor from different party | 0.017 |  | 0.011 |  | 0.006 |  |
|  | (0.016) |  | (0.016) |  | (0.018) |  |
| Difference | -0.021 |  | 0.001 |  | -0.002 |  |
|  | (0.022) |  | (0.023) |  | (0.027) |  |
| Electoral record and political controls | Yes |  | Yes |  | Yes |  |
| Economic controls | Yes |  | Yes |  | Yes |  |
| Economic controls x majority party | Yes |  | Yes |  | Yes |  |
| State fixed effects | Yes |  | Yes |  | Yes |  |
| State fixed effects x majority party | No |  | Yes |  | Yes |  |
| State fixed effects x governor's party | No |  | Yes |  | Yes |  |
| State fixed effects x majority party x governor's party | No |  | No |  | Yes |  |
| Year fixed effects | Yes |  | Yes |  | Yes |  |
| No. of states | 31 |  | 29 |  | 29 |  |
| No. of elections | 242 |  | 232 |  | 232 |  |
| Observations | 24,179 |  | 23,666 |  | 23,666 |  |

The table reports estimates of average marginal effects of changing the budget gridlock variable from zero to one on the probability of re-election for incumbent legislators. Each row reports the estimated average marginal effect for a particular type of incumbent. The rows labeled "Difference" report the differences in estimated marginal effects between the two rows immediately above. Standard errors (in parentheses) are estimated allowing for clustering at the state-year level.

The top part of the table focuses on the average partial effects of budget gridlock for majority party incumbents: We find a strong effect for this group when the governor belongs to the same party as the majority in the legislature. Ranging from -5 to -7 percentage points across the three columns, the estimates are quite stable and strongly statistically significant. Turning to the second row, the point estimates fall somewhere in the range between -2 and -4 percentage points when we condition on the governor belonging to a different party. While this is somewhat smaller than in the same-party case, the difference is in no case statistically significant.

The lower part of the table shows results for minority party members. Recall that the results in Table 2 showed no significant effect of budget gridlock on the probability of reelection for this group. The results in Table SI.2 show that this conclusion does not change when we also condition on membership or non-membership of the governor’s party. Comparing with the results for majority party incumbents, the difference between the two groups (not reported directly in Table SI.2) is around 5-8 percentage points when the governor belongs to the same party and around 4 percentage points when the governor belongs to a different party, and it is always statistically significant at the 5 percent level.

# Robustness checks of main analysis

Table A5 reproduces Table A4 above, but using linear regression models with incumbent vote shares as the dependent variable. Overall, the results are consistent with our main findings: Budget delays are associated with significantly lower vote shares for majority party incumbents, with coefficients on the budget gridlock variable of around 2 percentage points. For minority party incumbents, we also find negative coefficients, but they are numerically smaller and rarely statistically significant. We do not find any significant differences related to whether the incumbent belongs to the same party as the governor.

**Table A5: Budget gridlock and incumbent vote shares**

|  | (1) |  | (2) |  | (3) |  |
| --- | --- | --- | --- | --- | --- | --- |
|  | Vote share (percentage points) | | | | | |
|  |  |  |  |  |  |  |
| Majority party members, governor from same party | -2.31 |  | -2.39 |  | -2.37 |  |
|  | (1.06) |  | (1.08) |  | (1.28) |  |
| Majority party members, governor from different party | -2.13 |  | -2.31 |  | -2.35 |  |
|  | (0.96) |  | (0.99) |  | (1.10) |  |
| Difference | -0.18 |  | -0.08 |  | -0.02 |  |
|  | (1.29) |  | (1.37) |  | (1.70) |  |
| Minority party members, governor from same party | -1.59 |  | -1.00 |  | -2.19 |  |
|  | (0.92) |  | (0.94) |  | (0.84) |  |
| Minority party members, governor from different party | -1.24 |  | -0.84 |  | -0.54 |  |
|  | (0.94) |  | (0.88) |  | (1.13) |  |
| Difference | -0.36 |  | -0.16 |  | -1.65 |  |
|  | (1.19) |  | (1.20) |  | (1.45) |  |
| Electoral record and political controls | Yes |  | Yes |  | Yes |  |
| Economic controls | Yes |  | Yes |  | Yes |  |
| Economic controls x majority party | Yes |  | Yes |  | Yes |  |
| State fixed effects | Yes |  | Yes |  | Yes |  |
| State fixed effects x majority party | No |  | Yes |  | Yes |  |
| State fixed effects x governor's party | No |  | Yes |  | Yes |  |
| State fixed effects x majority party x governor's party | No |  | No |  | Yes |  |
| Year fixed effects | Yes |  | Yes |  | Yes |  |
| No. of states | 31 |  | 29 |  | 29 |  |
| No. of elections | 242 |  | 232 |  | 232 |  |
| Observations | 20,708 |  | 20,263 |  | 20,263 |  |

The table reports linear regression estimates of the partial effect of changing the budget gridlock variable from zero to one on the vote share for incumbent legislators. Each row reports the estimated effect for a particular type of incumbent. The rows labeled "Difference" report the differences in estimated effects between the two rows immediately above. Standard errors (in parentheses) are estimated allowing for clustering at the state-year level.

Table A6 shows results from a series of further robustness tests of our main analysis. We focus on our central result: The contrast in the electoral consequences of budget gridlock between majority vs. minority party incumbents. The baseline is the specification from column 4 of Table 2 (corresponding to Model 4 in Fig. 1), which is reproduced in column 1 of Table A6 for convenience. We only report marginal effects for outcome 1, reelection.

Columns 2 and 3 report results from specifications in which we have replaced our preferred measure of budget gridlock with a dummy variable that equals one if the state budget was delayed in any year between the previous and the current election for the state legislature (column 2), or a dummy variable that equals one if the state budget was delayed in the same year as the current election (column 3).^[[2]](#footnote-2)^ These alternative definitions of our key explanatory do not change the results much: The estimated marginal effects for majority party incumbents are slightly smaller than in the baseline specification, but they remain significantly negative and the difference compared to the estimated effects for minority party incumbents is still significant at the five percent level.

The baseline specification distinguishes between incumbent defeats to same-party challengers and defeats to challengers from other parties. As explained in section 3 of the main text, this means that we have to have to drop observations from multi-member districts where there is no unique successor to a defeated incumbent. In column (4) we report results from a model where these observations are retained in the sample. To do this, we pool all incumbent defeats in one base category and estimate a binomial logit model using a simple dummy for reelection as the dependent variable. The estimated marginal effects on the probability of reelection from this model are virtually identical to those from the baseline specification.

The analysis presented in the main text also includes electoral races in which the incumbent did not run for reelection, despite being eligible for it. As explained in section 3, these races are treated in the same way as elections in which the incumbent ran and lost. Column (5) shows that our conclusions are robust to altering this approach: This column reports estimates from a model that is identical to the baseline in column 1, but estimated on the subsample of electoral races in which the incumbent did in fact run for reelection. The results are again similar to the baseline.

We have also estimated our baseline model on the subsample of observations stemming from elections in which the same party controlled both chambers of the legislature, as well as on the subsample of observations for which the opposite was true. The results are reported in columns (6) and (7), respectively. Limiting the sample to elections following a cycle of unified party control of the state legislature does not change our results in any way, as the estimated marginal effects reported in column (6) are almost identical to those in the baseline analysis. When we limit the sample to elections following split-legislature cycles, standard errors increase somewhat due to the smaller sample size, but the point estimates of the marginal effects are again very similar to the baseline estimates.

Our data on budget timeliness suffers from a relatively large number of missing observations, especially in the early years of the analysis period (see Table A1 for details). We have estimated our model on the subsample of states for which we have non-missing information about budget timeliness in all years in the analysis period. The results of this exercise, shown in column (8), are very similar to the baseline results, suggesting that sample selection bias due to missing data on budget enactment dates is not an issue of concern.

Finally, we have estimated our model on the subsample of state elections in which the state unemployment rate was lower in the month of the election than it was 12 months prior to the election. We again find that budget gridlock lowers the reelection chances for majority party incumbents but has no significant effect for minority party incumbents. The difference between the two groups is less precisely estimated than in our baseline analysis (*p*-value of 0.075) but the point estimate is very similar in size.

# Calculating the aggregate effect of budget gridlock from micro-level estimates

To calculate the effect of changing the budget gridlock variable from zero to one on the majority party’s seat share from our micro-level estimates, we do as follows: from Table A2, Model 4, the probability of losing the seat to an opposite-party candidate increases by 3.5 percentage points for majority party incumbents and drops by 1.2 percentage points for minority party incumbents. For the electoral cycles included in our analysis sample, the average seat share held by the majority party in the lower house is 0.6. Hence, the average effect on the majority party’s seat share is -.035x0.6 -.012 x (1-0.6) = -.026. The 95% confidence interval - computed using the delta method – goes from 1.6 to 3.6.

# Analyzing aggregate election outcomes at the party-level

This section presents results from an analysis of election outcomes at the party level. The unit of analysis is an election for the lower house of the state legislature in a given state in a given year. As in our main analysis, we concentrate on elections held in the years between 1989 and 2007 in the 33 states that experienced at least one budget delay between 1988 and 2007. There are 283 such elections in total, but missing data on budget passage dates (predominantly in the early years) brings the number of elections in our analysis sample down to 261.^[[3]](#footnote-3)^

We estimate variants of the following linear model using ordinary least squares:

$ma{jshare}_{it}=\beta_{0}+\beta_{1}gridlock_{it}+\beta_{2}govsame_{it}+\beta_{3}{gridlock}_{it}*govsame_{it}+\mathbf{x}_{it}\boldsymbol{\delta+}\eta_{i}+\gamma_{t}+\epsilon_{it}$ (1)

The dependent variable is the seat share won in the lower-house election in state *i* in year *t* by the party that held a majority in the house before the election. Party seat shares are based on Carl Klarner’s data set on partisan balances in state legislatures (Klarner 2013). The key explanatory variable is *gridlock_it_*, the normalized number of legislative budget delays since the previous lower-house election described in section 3 of the main text. We include this variable directly, as well as interacted with the dummy variable *govsame*_it_, which takes the value one if the governor belongs to the same party as the majority in the lower house of the legislature in state *i* in year *t*. We also estimate a variant of the model in which *govsame* is interacted not only with the gridlock variable, but also with a number of economic control variables, as well as with the state fixed effects.

The control variables in **x**_it_ parallel those in the analysis of outcomes for individual incumbents: First, we include the seat share won by the current majority party in the previous election. Second, we control for gubernatorial and presidential coattails by including the major party vote share obtained by the candidate belonging to the same party as the majority in the state legislature in same-year gubernatorial/presidential elections, along with a dummy variable for gubernatorial election year. Also included is the presidential approval rating index described in section 3, now interacted with a dummy variable indicating whether the president belongs to the same party as the majority in the state legislature (this dummy is also included with no interaction). Third, we include the same economic controls as in the micro-level analysis: The one-year changes in the state unemployment rate and in real house prices, the change in the ratio of state government expenditures to GDP since the previous election, tax increases enacted since the previous election (in percent of general fund revenue), and the state government budget surplus (percent of state GDP) in the year of the election. State- and year fixed effects are included. Standard errors are computed allowing for clustering at the state level.

Table A7 presents regression estimates for the coefficients of main interest, i.e. $\beta_{1}$-$\beta_{3}$. Column (1) presents results for the same sample of elections as used in our micro-level analyses of election outcomes for individual incumbents. The coefficient on the budget gridlock variable is -.029 and significant at the 1 percent level. Column (2) extends the sample to include all 261 elections for which we have data, which does not change the results in any important way. We also use the extended sample in column (3), in which we add an interaction term between the budget gridlock variable and *govsame*_it_. The point estimates in this column suggest a budget gridlock effect on the majority party’s seat share of -.034 when the governor also belong to this party, versus -.021 in the opposite case, but the coefficient on the interaction term is not significant. Finally, column (4) adds interaction terms between *govsame*_it_ and the economic controls as well as state fixed effects. As in the micro-level analysis, adding such interaction terms makes the coefficient on the gridlock numerically larger, but the coefficient on the interaction term between this variable and *govsame*_it_ remains insignificant.

**Table A7: Budget gridlock and majority party seat shares, party-level analysis**

|  | (1) | (2) | (3) | (4) |
| --- | --- | --- | --- | --- |
|  | Dep. variable: Seat share obtained by majority party | | | |
| Budget gridlock | -0.029 | -0.027 | -0.021 | -0.034 |
|  | (0.010) | (0.010) | (0.011) | (0.012) |
| Governor from same party as majority in legislature | -0.004 | -0.002 | 0.003 | 0.044 |
|  | (0.008) | (0.008) | (0.011) | (0.026) |
| Budget gridlock x gov. from same party as majority in legislature |  |  | -0.013 | -0.007 |
|  |  |  | (0.019) | (0.026) |
| Political controls | Yes | Yes | Yes | Yes |
| Economics controls | Yes | Yes | Yes | Yes |
| Year fixed effects | Yes | Yes | Yes | Yes |
| State fixed effects | Yes | Yes | Yes | Yes |
| Economic controls x gov. same party | No | No | No | Yes |
| State fixed effects x gov. same party | No | No | No | Yes |
| No. of states | 31 | 33 | 33 | 31 |
| Observations | 242 | 261 | 261 | 251 |

The table reports regression results from estimation of equation (1). Column (1) reports results from an estimation on the same sample of 242 elections used in the micro-level analyses described in the main text. Columns (2) and (3) use the full sample of 261 elections for which we have sufficient information about budget gridlock, including elections in which all incumbents run in multi-member districts. Column (4) also includes elections with only multi-member districts, but the inclusion of interaction terms between *govsame* and state fixed effects implies that elections in Delaware and Rhode Island must be omitted from the sample to avoid perfect collinearity. Standard errors (in parentheses) are estimated allowing for clustering at the state level.

1. The simultaneous inclusion of interaction terms between state fixed effects and both party affiliation dummies requires estimation of a separate state fixed effect for each combination of majority party membership and membership of the governor’s party. For this reason, we drop Delaware from the analysis. The reason is that the governor of Delaware belonged to the same party as the majority in the state legislature in all of the years in our analysis period, implying that majority party membership is inseparable from membership of the governor’s party. [↑](#footnote-ref-1)
2. In states with biennial budgets that are passed in non-election years, the dummy is equal to one if the budget was delayed in the year before the election. [↑](#footnote-ref-2)
3. In contrast to the individual-level analysis, we do have data on state-level election outcomes for all elections held in New Jersey and North Dakota in the analysis period. This adds 19 elections to the analysis sample. [↑](#footnote-ref-3)
